# Genome-Scale Screening and Combinatorial Optimization of Gene Overexpression Targets to Improve Cadmium Tolerance in *Saccharomyces cerevisiae*

**DOI:** 10.3389/fmicb.2021.662512

**Published:** 2021-07-14

**Authors:** Yongcan Chen, Jun Liang, Zhicong Chen, Bo Wang, Tong Si

**Affiliations:** ^1^CAS Key Laboratory of Quantitative Engineering Biology, Shenzhen Institute of Synthetic Biology, Shenzhen Institute of Advanced Technology, Chinese Academy of Sciences, Shenzhen, China; ^2^Southern Marine Science and Engineering Guangdong Laboratory (Guangzhou), Shenzhen, China

**Keywords:** gene overexpression, combinatorial optimization, genome-scale engineering, *Saccharomyces cerevisiae*, cadmium tolerance

## Abstract

Heavy metal contamination is an environmental issue on a global scale. Particularly, cadmium poses substantial threats to crop and human health. *Saccharomyces cerevisiae* is one of the model organisms to study cadmium toxicity and was recently engineered as a cadmium hyperaccumulator. Therefore, it is desirable to overcome the cadmium sensitivity of *S. cerevisiae via* genetic engineering for bioremediation applications. Here we performed genome-scale overexpression screening for gene targets conferring cadmium resistance in CEN.PK2-1c, an industrial *S. cerevisiae* strain. Seven targets were identified, including *CAD1* and *CUP1* that are known to improve cadmium tolerance, as well as *CRS5, NRG1, PPH21, BMH1*, and *QCR6* that are less studied. In the wild-type strain, cadmium exposure activated gene transcription of *CAD1*, *CRS5*, *CUP1*, and *NRG1* and repressed *PPH21*, as revealed by real-time quantitative PCR analyses. Furthermore, yeast strains that contained two overexpression mutations out of the seven gene targets were constructed. Synergistic improvement in cadmium tolerance was observed with episomal co-expression of *CRS5 and CUP1*. In the presence of 200 μM cadmium, the most resistant strain overexpressing both *CAD1* and *NRG1* exhibited a 3.6-fold improvement in biomass accumulation relative to wild type. This work provided a new approach to discover and optimize genetic engineering targets for increasing cadmium resistance in yeast.

## Introduction

Heavy metal contamination is a severe environmental problem (Vareda et al., 2019; Hu et al., 2020). Cadmium is one of the primary risks to human health due to the pollution of food crops and drinking water (Nordberg et al., 2018). *Saccharomyces cerevisiae* is a model organism to study cadmium toxicity, and related mechanisms include glutathione biosynthesis, stress response, vacuole transportation and sorting, metal ion homeostasis, and chromatin remodeling (Wysocki and Tamas, 2010). Notably, cadmium tolerance mechanisms are conserved between *S. cerevisiae* and plants, including gene overexpression of glutathione reductase (Kim et al., 2012), metallothionein (MT) (Wei et al., 2016; Ansarypour and Shahpiri, 2017), and phytochelatin (Cahoon et al., 2015; Yang et al., 2017).

Physiochemical and biological remediation are common strategies to treat heavy metal contamination. For example, chemical precipitation is frequently utilized, but the main limitations include secondary waste generation and complex infrastructure requirements (Fu and Wang, 2011; Li et al., 2019; Vardhan et al., 2019). Phytoremediation by “hyperaccumulator” plants is a promising alternative, but it suffers from the long life cycle and engineering challenges of plants (Rascio and Navari-Izzo, 2011; Reeves et al., 2018). On the other hand, microbial bioremediation may provide a scalable and cost-effective solution. In particular, *S. cerevisiae* has been recently engineered as a heavy-metal hyperaccumulator when equipped with selected membrane transporters and enhanced vacuolar compartmentalization (Sun et al., 2019, 2020). Furthermore, *S. cerevisiae* can be utilized to ferment the biomass of phytoremediation plants (Jing et al., 2020). However, *S. cerevisiae* is relatively sensitive to cadmium, and it is desirable to improve its cadmium robustness of *S. cerevisiae* as a future bioremediation agent.

Chemical tolerance is a complex phenotype with hundreds of genetic determinants (Almeida et al., 2007). Due to the lack of mechanistic understanding, it often requires genome-wide screening to identify mutations that confer resistance (Warner et al., 2010; Si et al., 2015b; Chen et al., 2020; Liu et al., 2020). For such endeavors, gene-deletion strain libraries are often utilized in *S. cerevisiae*, but most identified mutations lead to cadmium sensitivity (Jin et al., 2008; Ruotolo et al., 2008; Serero et al., 2008; Thorsen et al., 2009). Genetic overexpression libraries are less studied, and hence beneficial mutations are relatively scarce (Hwang et al., 2009; Fang et al., 2016). Notably, existing screening campaigns for cadmium resistance are confined to certain laboratory strains derived from the S288c background. Moreover, only single genetic mutations are investigated, whereas combinatorial optimization of multiplex gene targets is often needed for resistance engineering (Si et al., 2015a; Jiang et al., 2020).

Here we performed genome-wide screening in *S. cerevisiae* using a comprehensive cDNA plasmid library that was previously constructed (Si et al., 2017). We utilized a widely used industrial strain, CEN.PK2-1c, whose cadmium resistance has not been studied. Both known and new gene targets were identified to confer increased resistance. Further improvement in cadmium tolerance was achieved via the combinatorial introduction of individual mutations. Dosage-dependence and genetic interactions were also observed among different gene targets.

## Materials and Methods

### Strains, Media, and Cultivation Conditions

A previously reported derivative (CAD) of CEN.PK2-1c (*MATa ura3-52 trp1-289 leu2-3,112 his3D1 MAL2-8C SUC2*) was utilized in this study as the wild-type (WT) background, harboring an integrated RNA-interference pathway (Si et al., 2015a). Overexpression cassettes were either integrated into the genomic *LEU2* locus or cloned in episomal plasmids (Supplementary Tables 1, 2). *Saccharomyces cerevisiae* strains were cultivated in either synthetic dropout medium (0.17% Difco yeast nitrogen base without amino acids and ammonium sulfate, 0.5% ammonium sulfate and 0.083% amino acid dropout mix, 0.01% adenine hemisulfate, and 2% glucose) or YPAD medium (1% yeast extract, 2% peptone, 0.01% adenine hemisulfate, and 2% glucose). For cadmium stresses, 100 mM Cd(NO_3_)_2_ solution stock was added to the above medium to indicated final concentrations. All media were adjusted to pH = 4.5. *Saccharomyces cerevisiae* strains were cultured at 30°C and with 250 r.p.m. agitation in culture tubes or 96-well microplates. Cadmium nitrate tetrahydrate was purchased from Aladdin (C118495). Other chemicals were purchased through Sigma-Aldrich.

### Genome-Scale Overexpression Screening

The overexpression library was constructed by cloning the normalized cDNA library into a single-copy plasmid pRS416, which contains a P_TEF1_ promoter and a T_PGK1_ terminator (Si et al., 2017). The library was transformed into CAD strain by the standard LiAc/ssDNA/PEG protocol (Gietz and Schiestl, 2007) with an optimized condition, where 15 μg plasmid DNA was used to transform 30 OD_600_(optical density at 600 nm) unit competent yeast cells by heat shock at 42°C for 1 h. Cell transformant suspension was diluted 10-fold and spread onto SC-Ura agar plates containing 200 μM Cd(NO_3_)_2_. Approximately 1.5 × 10^6^ independent yeast clones were obtained. The WT strain containing an empty pRS416 vector was used as a control. Clones with substantially larger colony sizes relative to WT were selected for further characterization. The overexpression cassettes of isolated plasmids were subjected to Sanger sequencing and Basic Local Alignment Search Tool (BLAST) analysis for target identification. Plasmid retransformation into a fresh WT background was performed to confirm positive targets.

### Cadmium Tolerance Assays

Yeast strain cultures transformed with the empty vector or gene-overexpressing plasmids were inoculated and cultivated to the early stationary phase. OD_600_ values were measured using a Ultrospec 10 cell density meter (Biochrom, 80-2116-30). For spot assay, one OD_600_ unit of yeast cells was serially diluted at tenfold, spotted (3 μL) on SC-Ura agar supplemented with 200 μM Cd(NO_3_)_2_, and incubated at 30°C for 2 to 3 days. For microculture growth assay, growth phase-synchronized cultures were diluted to an initial OD_600_ of 0.2 in 200 μL of SC-Ura, SC-Ura/Leu or SC-Ura/Leu/Trp medium supplemented with indicated Cd(NO_3_)_2_ concentrations and cultivated at 30°C in 96-well, round-bottom plates (Corning). A BioTek Epoch2 microplate reader was utilized to monitor OD_600_ at 30 min intervals. The OD_600_ values at 48 h from three independent experiments were used to calculate relative cadmium tolerance [Equation (1), see below].

### Real-Time, Quantitative PCR (RT-qPCR) Analyses

The mid-log phase cell cultures of the WT strain were treated with 50, 100, or 200 μM Cd(NO_3_)_2_ for 2 h. Total RNA was isolated using a Bacteria RNA Extraction Kit (Vazyme, R403-01), and 1 μg total RNA was reverse transcribed using HiScript III RT SuperMix for qPCR with gDNA wiper (Vazyme, R323-01). RT-qPCR reaction and data analysis were performed in an Analytik Jena qTOWER3 Real-Time PCR Thermal Cycler using ChamQ Universal SYBR qPCR Master Mix (Vzyme, Q711-02) following the manufacturer’s instructions. Primers for qPCR reactions were listed in Supplementary Table 3.

### Dual-Mutation Strain Construction

The identified overexpression cassettes of cadmium-tolerant genes were PCR amplified with the TEF1p and PGK1t primers and cloned into the pRS415 vector using the Gibson Assembly Cloning Kit (New England Biolabs) (Supplementary Table 2). The primers used are listed in Supplementary Table 3. For genomic integration, the expression cassettes and *LEU2* marker gene were PCR amplified with primers fusing with 40 bp homology arms and integrated into the *LEU2* locus via homologous recombination.

### Calculation of Relative Cadmium Tolerance and Combinatorial Effect Scores

The final OD_600_ at 48 h was used to calculate relative cadmium tolerance using Equation (1):

(1) $$CadmiumTolerance\left(CT_{G}\right)=\frac{OD_{G,T}/OD_{G,C}}{OD_{V,T}/OD_{V,C}}$$

where *G* indicates a gene-overexpression target and *V* means the empty-vector control. *T* indicates cadmium treatment and *C* means the cadmium-free control. A value larger than 1 indicates improved cadmium tolerance relative to WT. The combinatorial effects of cadmium-tolerant genes were calculated using Equation (2) (Anastassiou, 2007):

(2) $$CombinatorialEffect\left(CE_{G1,G2}\right)=\left(CT_{G1,G2}-1\right)-\left[\left(CT_{G1}-1\right)\left(CT_{G2}-1\right)\right]$$

where *CT*_G1,G2_ indicates cadmium tolerance of the strain co-expressing genes 1 and 2, whereby *CT*_G1_ and *CT*_G2_ means cadmium tolerance of the strain overexpressing only gene 1 or gene 2, respectively. A positive value of *CE*_G1,G2_ indicates synergistic effect.

## Results

### Genome-Wide Overexpression Screening for Enhanced Cadmium Resistance

**FIGURE 1:**
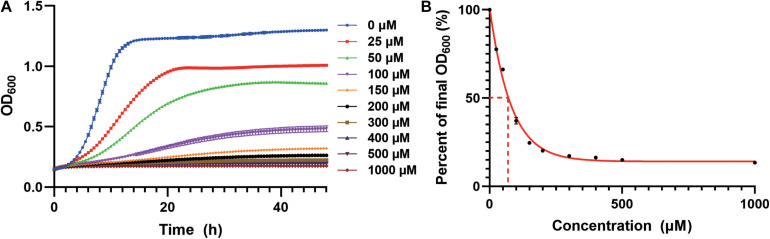
Growth inhibition by cadmium in the WT strain. **(A)** Cd(NO_3_)_2_ was added to the SC-Ura medium at indicated concentrations. Cellular growth was monitored every 30 min for 48 h as optical density at 600 nm (OD_600_). **(B)** Cadmium concentration-dependent reduction in the normalized final OD_600_ at 48 h. The curve was best fitted using a one-phase exponential decay model. Dash lines indicated the cadmium concentration for 50% growth inhibition.

We first studied the cadmium sensitivity of an industrial yeast strain CEN.PK2-1c using a previously constructed derivative (Si et al., 2015a). The WT strain transformed with the empty plasmid (pRS416) was cultivated in the synthetic dropout medium containing Cd(NO_3_)_2_ as a source of cadmium. We observed growth inhibition in a cadmium concentration-dependent manner (Figure 1A), and a 50% decrease in total biomass accumulation was elicited by approximately 70 μM cadmium (Figure 1B). Nearly complete growth inhibition was observed at 200 μM cadmium, which was selected to screen for resistant strains (Figures 1A,B). Similarly, the lethal dose of cadmium for BY4741 yeast strain, an S288c derivative, was 150 μM (Kuang et al., 2015).

**FIGURE 2:**
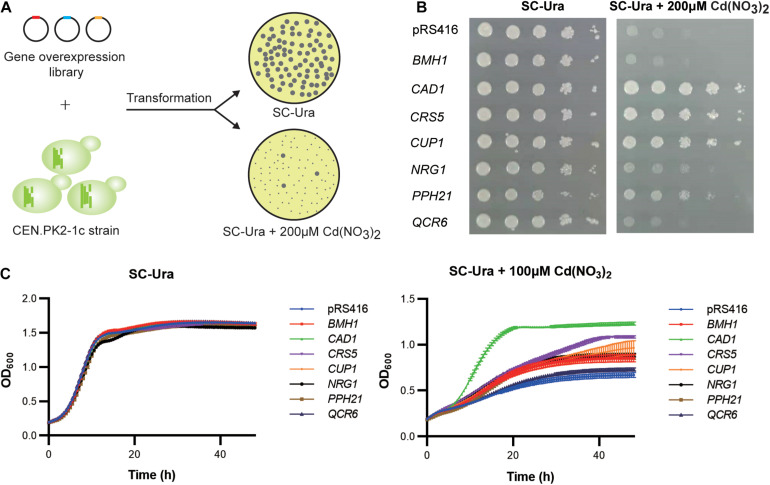
Genome-scale screening for cadmium-tolerant genes. **(A)** Scheme of genome-wide overexpression screening for cadmium resistant clones. **(B)** Spot assay on agar media. Yeast cultures were serially diluted, spotted, and cultivated for 2–3 days on agar plates with or without 200 μM cadmium. **(C)** Time-course of cellular growth in liquid media. The time-course of OD_600_ was collected at 30 min intervals for 48 h with or without 100 μM cadmium. Error bars indicate standard deviations of three biological replicates.

We transformed the WT strain with a genome-scale library on the pRS416 vector, which was previously constructed based on a normalized, full-length enriched cDNA library of *S. cerevisiae* (Si et al., 2017). Over 10^6^ independent transformants were obtained to ensure sufficient coverage of >92% of all yeast genes (Si et al., 2017). Resistant clones were selected based on colony sizes (Figure 2A). Plasmid DNA sequencing of the 160 largest colonies revealed 95 (59.4%), 27 (16.9%), and 5 (3.13%) clones contained the upregulation cassettes of *CUP1*, *CAD1*, and *PPH21* genes, respectively. After retransformation into a fresh WT background, seven gene targets were confirmed with resistant phenotypes to 100 μM cadmium in liquid media, including *CAD1*, *CRS5*, *CUP1*, *NRG1*, *PPH21*, *BMH1*, and *QCR*6 (Figure 2C). Except for *BMH1* and *QCR6*, gene overexpression of all other targets also conferred enhanced growth on agar media containing 200 μM cadmium (Figure 2B; Supplementary Figure 1). Gene Ontology (GO) analysis suggested that these genes are involved in stress response to chemical, oxidative, osmotic and DNA damage, regulation of transcription, translation, protein modification and cell cycle, and organization of organelle, cell wall and vacuole (Supplementary Table 4), which are all relevant mechanisms to cadmium toxicity.

### Gene Regulation of Identified Targets in Response to Cadmium Stress

**FIGURE 3:**
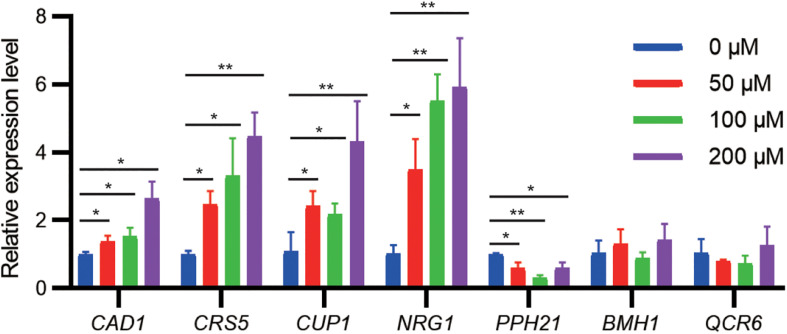
qPCR analysis of cadmium-tolerant genes upon cadmium stress. During the exponential growth phase, the WT strain was treated with Cd(NO_3_)_2_ at indicated concentrations for 2 h. Error bars indicate standard derivations of three biological replicates. *P < 0.05; **P < 0.01.

To examine whether gene expression of identified targets is regulated by cadmium stress, we challenged the WT strain at the exponential growth phase with different cadmium concentrations. qPCR results indicated that the mRNA levels of *CAD1*, *CRS5*, *CUP1*, and *NRG1* were significantly upregulated by cadmium in a concentration-dependent manner (Figure 3). Genetic upregulation of *CRS5*, *CUP1*, and *NRG1* by cadmium was consistent with previous transcriptomic studies in the S288c background (Jeyaprakash et al., 1991; Fauchon et al., 2002; Jin et al., 2008). On the other hand, cadmium treatment suppressed *PPH21* expression, and no significant changes in mRNA levels were observed for *BMH1* and *QCR6* (Figure 3). Consistently, previous studies did not report substantial transcriptional alterations in *PPH21*, *BMH1*, or *QCR6* upon cadmium treatment (Jeyaprakash et al., 1991; Fauchon et al., 2002; Jin et al., 2008).

### Combinatorial Optimization of Cadmium-Tolerant Genes

**FIGURE 4:**
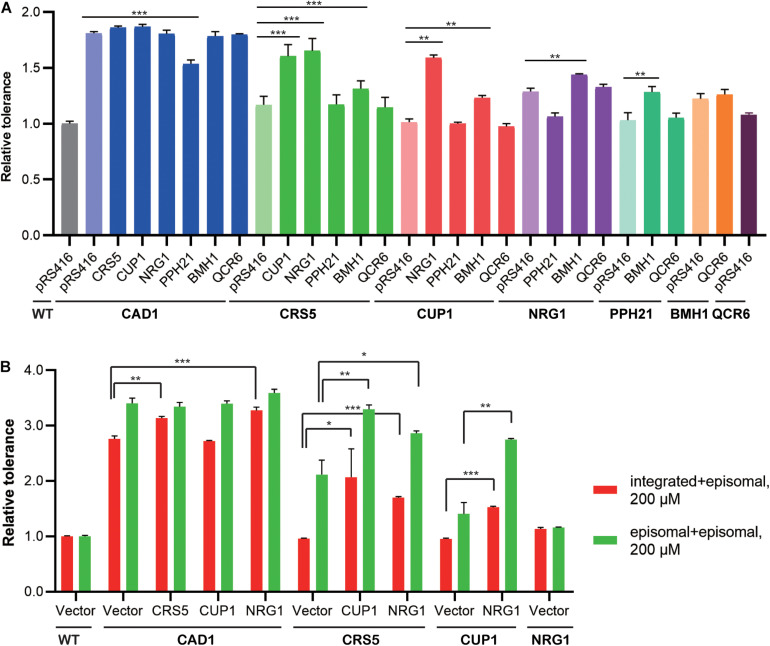
Combinatorial effects of cadmium-tolerant genes. Relative cadmium tolerance of the engineered strains with episomal plasmids in the presence of 100 μM **(A)** or 200 μM **(B)** cadmium was calculated as described above in Materials and Methods. **(A)** Genomic and episomal expression are labeled as bold and italic, respectively. **(B)** Red bars indicate overexpression from genomic (under the line) and episomal (above the line) vectors, and green bars indicate episomal expression for both targets. Values are means and standard derivations (*n* = 3). *P < 0.05; **P < 0.01; ***P < 0.001.

We then constructed mutant strains harboring two overexpression targets to improve cadmium tolerance (Figure 4A; Supplementary Figure 2). First, each identified cassette was separately integrated at the *LEU2* locus of the yeast genome. In the presence of 100 μM cadmium, genomic and episomal overexpression of *CAD1*, *NRG1*, *BMH1*, or *QCR6* conferred comparative improvement in cadmium tolerance relative to WT (Supplementary Figure 3). On the contrary, the strains integrated with *CRS5*, *CUP1*, or *PPH21* cassettes showed substantially weaker cadmium resistance improvement relative to their plasmid-overexpression counterparts (Supplementary Figure 3). Differences in gene expression levels were previously noted between genomic integration and episomal expression (Crook et al., 2014; Si et al., 2015a). Therefore, genomic integration (1 per cell) of the *CUP1* cassette may result in a lower overexpression level compared with pRS416 (3-4 copies per cell), so that a minimum threshold of *CUP1* overexpression required for cadmium resistance was not achieved (Jeyaprakash et al., 1991; Adamo et al., 2012). To further confirm dosage effects, we constructed yeast strains overexpressing a single-gene target on pRS426, which is a 2 μ multicopy plasmid (20–80 copies per cell). Indeed, cadmium tolerance of *CUP1*-overexpressing yeast strains showed a pRS426 > pRS416 > integration pattern. No substantial dosage effects were observed for other targets except for *CRS5* (Supplementary Figures 3, 4). Notably, both *CUP1* and *CRS5* encode yeast metallothionein, which suggested a possibly shared mechanism.

Dual overexpression was then achieved by transforming the above-mentioned *LEU2*-integrated strains (Figure 4A, **bold**) with overexpression plasmids (Figure 4A, *italic*). Only selected combinations were constructed to save experimental efforts, whereby a more potent target was integrated into the yeast genome, and a weaker target was placed on the episomal plasmid. In the presence of 100 μM cadmium, *CAD1*-integrated strains generally exhibited high levels of resistance, except when transformed with a *PPH21*-overexpressing plasmid. Mutant strains harboring *CRS5-CUP1*, *CRS5-NRG1*, and *CUP1-NRG1* combinations also showed robust resistance. Therefore, *CAD1*, *CRS5*, *CUP1*, and *NRG1* were selected for further optimization, and new strains with two targets both expressed from episomal plasmids (pRS415 and pRS416) were constructed. Mutant strains containing integration-plasmid and plasmid-plasmid combinations were evaluated for the time-course of cellular growth under 200 μM cadmium (Figure 4B; Supplementary Figure 5). The highest tolerance was achieved by *CAD1* and *NRG1* co-expression both from plasmids, and the corresponding strain exhibited a 3.6-fold improvement in the final OD_600_ relative to WT. Unfortunately, no further improvement was achieved with triple overexpression of *CAD1-NRG1-CRS5* or *CAD1-NRG1-CUP1* from episomal plasmids (pRS414, pRS415, and pRS416) (Supplementary Figure 6).

Also, we examined the combinatorial effects among identified gene targets. Genetic interactions were evaluated using Equation (2) as previously reported, based on relative tolerance values calculated by Equation (1) (see Materials and Methods). In the presence of 100 μM cadmium, synergistic effects were observed between genome-integrated *CRS5* and plasmid-borne *NRG1*, as well as between genome-integrated *CUP1* and plasmid-borne *NRG1* (Figure 4A). In the presence of 200 μM cadmium, synergistic interaction was noted between *CRS5* and *CUP1* when both expressed episomally (Figure 4B). Synergistic improvement confirmed the necessity for multiplex, combinatorial optimization of individual engineering targets (Wang et al., 2009; Si et al., 2015a, 2017; Cao et al., 2020; Chen et al., 2020).

## Discussion

Here we performed the first genome-wide overexpression screening to engineer cadmium resistance in an industrial CEN.PK-derived *S. cerevisiae* strain. Possible mechanisms underlying identified targets may be deduced from GO analysis (Supplementary Table 4) and known functions reported in literature (Supplementary Table 5). Particularly, identifying well-established targets such as *CAD1* and *CUP1* indicated our approach was effective. These results also suggested that the corresponding mechanisms are conserved among different *S. cerevisiae* backgrounds for cadmium tolerance. For example, *CAD1* encodes an AP-1-like basic leucine zipper (bZIP) transcriptional activator, which increased cadmium resistance when expressed on a multicopy plasmid (Wu et al., 1993; Hirata et al., 1994). Cad1p was imported to the nucleus from cytoplasm upon cadmium stress, upregulating *FRM2* for oxidative stress attenuation, as well as activating gene expression of *SLT2*, *RLM1*, and *CHS1* for cell-wall maintenance (Fernandes et al., 1997; Bilsland et al., 2004; Azevedo et al., 2007; Mazzola et al., 2015). In addition to nuclear import, our qPCR results implied that upregulation of *CAD1* induced by cadmium may also participate in these processes (Figure 3). Moreover, we confirmed another known target, *CUP1*, which was reported to increase copper and cadmium resistance when constitutively expressed on high-copy episomal plasmids (Ecker et al., 1986).

Several less studied overexpression targets were also isolated. For example, Nrg1p is a Rim101-mediated transcriptional repressor. *NRG1* overexpression enhanced transcriptional repression of *TAT1*, whose deletion also increased cadmium tolerance (Ruotolo et al., 2008) (Supplementary Table 5). Moreover, *PPH21* encodes a catalytic subunit of protein phosphatase 2A (PP2A), which mediates cadmium-induced repression of class I transcription by targeting the formation/dissociation of the Pol I-Rxn3 complex in yeast (Zhou et al., 2013). It was reported that PP2A homolog prevented cadmium-induced cell death via inactivation of Erk1/2 and JNK and participated in MT expression regulation through dephosphorylation of MTF-1 in mammalian cells (Chen et al., 2008, 2014) (Supplementary Table 5). Also, *BMH1* encodes a member of 14-3-3 proteins that function in various biological processes such as stress response (Kumar, 2017), and yeast cells expressing the human 14-3-3β/α showed enhanced cadmium resistance (Clapp et al., 2012) (Supplementary Table 5).

Notably, two new overexpression targets were identified in this study. We demonstrated plasmid-overexpression of *CRS5* increased cadmium tolerance in *S. cerevisiae* for the first time (Figures 2B,C). *CRS5* and *CUP1* both encode MT in *S. cerevisiae* (Chatterjee et al., 2020), and Crs5p possesses a Cd(II)-binding ability (Pagani et al., 2007) (Supplementary Table 5). Gene deletion of *crs5* did not lead to cadmium sensitivity (Culotta et al., 1994). Like *CUP1*, genomic overexpression of *CRS5* was less effective than episomal expression (Supplementary Figures 3, 4). In addition, both *CUP1* and *CRS5* transcription levels were upregulated upon cadmium exposure (Figure 3), suggesting dosage effects of MT may be common when regarding cadmium tolerance (Jeyaprakash et al., 1991; Adamo et al., 2012). Furthermore, *QCR6* encodes the subunit 6 of the ubiquinol cytochrome-c reductase complex (complex III). Complex III is a component of the mitochondrial inner membrane electron transport chain, which is suggested as a substantial source of reactive oxygen species (ROS) induced by cadmium in mammalian tissues (Wang et al., 2004) (Supplementary Table 5). Here we showed *QCR6* overexpression weakly increased cadmium tolerance in yeast (Figures 2B,C).

In addition to single targets, we also explored multiplex overexpression optimization for improving cadmium resistance in *S. cerevisiae*. The most resistant strain overexpressing both *CAD1* and *NRG1* on plasmids exhibited a 3.6-fold improvement in biomass accumulation compared with WT under 200 μM cadmium, and 1.1-fold and 3.1-fold improvement relative to the mutant strains containing individual overexpression of *CAD1* and *NRG1*, respectively. Furthermore, additive and synergistic effects were observed among overexpression targets. Additive effects for *NRG1* and *BMH1* were observed in *CRS5*-, *CUP1*-, *NRG1*-, or *PPH21*-integrated strains (Figure 4), possibly mediated *via* the cooperation of different tolerant mechanisms. For example, the gene products of *CRS5*, *CUP1*, *NRG1*, and *PPH21* are metal-binding proteins, whereby *NRG1* and *BMH1* both encode transcription factors (Supplementary Table 4). Synergistic tolerance improvement was achieved by *CRS5-NRG1*, *CUP1-NRG1*, and *CRS5-CUP1* co-expression (Figure 4), suggesting the presence of genetic interactions that warrant future mechanistic investigation.

Certain discrepancies were observed between this study and previous literature. For example, overexpression of *PPH21* improved cadmium tolerance (Figure 2), but it was down regulated in response to cadmium stress (Figure 3). It is not surprising, however, because the genes with substantially altered transcription and translation revealed by transcriptomic and proteomic profiling do not necessarily represent effective targets for genetic engineering (Momose, 2001; Vido et al., 2001; Fauchon et al., 2002; Jin et al., 2008; Ruotolo et al., 2008; Serero et al., 2008; Thorsen et al., 2009; Huang et al., 2016; Marmiroli et al., 2016). Indeed, little overlap was observed between transcriptomics and genome-deletion screening results for cadmium sensitivity (Serero et al., 2008). Moreover, different sets of gene targets were often identified during different genome-wide screening campaigns targeting cadmium resistance (Thorsen et al., 2009), and the main reasons may include variations in strain background, screening conditions, and the formats of genetic mutations. For example, we observed different effects of the same gene target between genomic and episomal expression (Supplementary Figure 3), as well as between liquid and agar media (Figure 2). Therefore, it is necessary to perform genome-wide screening and multiplex optimization in a particular target strain under conditions that best reflect the real-world application scenario. Considering a range of 1–100 mg/L (∼8.9–890 μM) cadmium in wastewater (Huang et al., 2020), existing efforts on engineering cadmium-resistant yeast for bioremediation has only achieved moderate success. For example, upon heterologous introduction of TaPCS1 that encodes a phytochelatin synthase, *S. cerevisiae* was engineered as a plant-like cadmium hyperaccumulator in the presence of around 100 μM cadmium (Sun et al., 2019). In this study, we engineered a yeast strain that can tolerate up to 400 μM cadmium by co-overexpression of *CAD1* and *NRG1* (Supplementary Figure 6). But further endeavor is warranted to further improve the resistance to cadmium and other inhibitors in waste water for real-world applications using *S. cerevisiae*.

Another limitation of the current approach is the inability to explore functional potential within the *S. cerevisiae* pan-genome due to the use of a cDNA-derived library of a select yeast strain. For example, *PCA1* encodes a cadmium efflux transporter. Whereas some natural *S. cerevisiae* isolates harbor a functional *PCA1* gene that confers cadmium resistance, most laboratory *S. cerevisiae* strains including CEN.PK2-1c used in this study contain a nonfunctional Pca1p due to a G970R missense mutation (Adle et al., 2007; Wei et al., 2014; Wong et al., 2021). Therefore, it is unlikely to identify *PCA1* as a positive hit via genome-scale screening using a CEN.PK cDNA-derived overexpression library. Moreover, the cDNA-templated library can only cover endogenous genes. To engineer CEN.PK yeast strains as bioremediation agents, it is necessary to screen heterologous genes to further improve its cadmium tolerance in future studies.

## Conclusion

Using a genome-wide approach, we identified new and known targets and their dual combinations that substantially improved cadmium resistance of an industrial *S. cerevisiae* strain. Genome-wide search and optimization of more than two targets are desirable and underway for achieving superior cadmium robustness via iterations of the current workflow either manually (Si et al., 2015a) or using an automated biofoundries (Si et al., 2017). This workflow can also be extended to engineer robustness toward other chemical inhibitors in *S. cerevisiae*.

## Data Availability Statement

The original contributions presented in the study are included in the article/Supplementary Material, further inquiries can be directed to the corresponding authors.

## Author Contributions

YC, TS, and BW conceived this study. YC designed and performed genome-scale screening, gene expression analysis, combinatorial optimization, and drafted the manuscript. JL measured growth inhibition of the WT strain by cadmium. ZC assisted with mutant screening and confirmation. TS and BW supervised the research. All authors proofread the manuscript before submission.

## Conflict of Interest

The authors declare that the research was conducted in the absence of any commercial or financial relationships that could be construed as a potential conflict of interest.
