# Supplementary material for: Genome-Scale Screening and Combinatorial Optimization of Gene Overexpression Targets to Improve Cadmium Tolerance in Saccharomyces cerevisiae
Source: Front Microbiol. 2021 Jul 14;12:662512. doi: 10.3389/fmicb.2021.662512 (PMC8318699; doi:10.3389/fmicb.2021.662512)
Supplement: Supplementary file 1 [file Data_Sheet_1.docx]

**SUPPLEMENTARY MATERIAL**

**Supplementary Table 1.** **List of *S. cerevisiae* strains in this study**

| **Strain** | **Genotype** | **Reference** |
| --- | --- | --- |
| CEN.PK2-1c | *MATa ura3-52 trp1-289 leu2-3,112 his3Δ1 MAL2-8C SUC2* | (Si et al., 2015) |
| CAD | *MATa δ::*P_TEF1_-*ago1*-P_TPI1_-*dcr1* | (Si et al., 2015) |
| CAD_CAD1 | *MATa δ::*P_TEF1_-*ago1*-P_TPI1_-*dcr1 leu2::LEU2-*P_TEF1_*-CAD1* | This study |
| CAD_CRS5 | *MATa δ::*P_TEF1_-*ago1*-P_TPI1_-*dcr1 leu2::LEU2-*P_TEF1_*-CRS5* | This study |
| CAD_CUP1 | *MATa δ::*P_TEF1_-*ago1*-P_TPI1_-*dcr1 leu2::LEU2-*P_TEF1_*-CUP1* | This study |
| CAD_NRG1 | *MATa δ::*P_TEF1_-*ago1*-P_TPI1_-*dcr1 leu2::LEU2-*P_TEF1_*-NRG1* | This study |
| CAD_PPH21 | *MATa δ::*P_TEF1_-*ago1*-P_TPI1_-*dcr1 leu2::LEU2-*P_TEF1_*-PPH21* | This study |
| CAD_BMH1 | *MATa δ::*P_TEF1_-*ago1*-P_TPI1_-*dcr1 leu2::LEU2-*P_TEF1_*-BMH1* | This study |
| CAD_QCR6 | *MATa δ::*P_TEF1_-*ago1*-P_TPI1_-*dcr1 leu2::LEU2-*P_TEF1_*-QCR6* | This study |

**Supplementary Table 2. List of plasmids used in this study**

| **Plasmid** | **Primers for PCR** | **Template for PCR** | **Vector/linearization enzymes** | **Notes** |
| --- | --- | --- | --- | --- |
| pRS416-P_TEF1_-CAD1-T_PGK1_ | Plasmids isolated from selected clones on SC-Ura plates in cadmium tolerance screening | | | Constructions of expression cassettes for corresponding cadmium-tolerant genes;  Helper plasmids to clone expression cassettes into the pRS415 vector |
| pRS416-P_TEF1_-CRS5-T_PGK1_ |  |  |  |  |
| pRS416-P_TEF1_-CUP1-T_PGK1_ |  |  |  |  |
| pRS416-P_TEF1_-NRG1-T_PGK1_ |  |  |  |  |
| pRS416-P_TEF1_-PPH21-T_PGK1_ |  |  |  |  |
| pRS416-P_TEF1_-BMH1-T_PGK1_ |  |  |  |  |
| pRS416-P_TEF1_-QCR6-T_PGK1_ |  |  |  |  |
| pRS415-P_TEF1_-CAD1-T_PGK1_ | TEF1p For/PGK1t Rev | pRS416-P_TEF1_-CAD1-T_PGK1_ | pRS415 by *XhoI*/*BamHI* | Construction of expression cassettes for corresponding cadmium-tolerant genes;  Helper plasmids to integrate expression cassettes into *leu2* site |
| pRS415-P_TEF1_-CRS5-T_PGK1_ |  | pRS416-P_TEF1_-CRS5-T_PGK1_ |  |  |
| pRS415-P_TEF1_-CUP1-T_PGK1_ |  | pRS416-P_TEF1_-CUP1-T_PGK1_ |  |  |
| pRS415-P_TEF1_-NRG1-T_PGK1_ |  | pRS416-P_TEF1_-NRG1-T_PGK1_ |  |  |
| pRS415-P_TEF1_-PPH21-T_PGK1_ |  | pRS416-P_TEF1_-PPH21-T_PGK1_ |  |  |
| pRS415-P_TEF1_-BMH1-T_PGK1_ |  | pRS416-P_TEF1_-BMH1-T_PGK1_ |  |  |
| pRS415-P_TEF1_-QCR6-T_PGK1_ |  | pRS416-P_TEF1_-QCR6-T_PGK1_ |  |  |
| pRS426-P_TEF1_-CAD1-T_PGK1_ |  | pRS416-P_TEF1_-CAD1-T_PGK1_ | pRS426 by *XhoI*/*BamHI* | Construction of expression cassettes for corresponding cadmium-tolerant genes |
| pRS426-P_TEF1_-CRS5-T_PGK1_ |  | pRS416-P_TEF1_-CRS5-T_PGK1_ |  |  |
| pRS426-P_TEF1_-CUP1-T_PGK1_ |  | pRS416-P_TEF1_-CUP1-T_PGK1_ |  |  |
| pRS426-P_TEF1_-NRG1-T_PGK1_ |  | pRS416-P_TEF1_-NRG1-T_PGK1_ |  |  |
| pRS426-P_TEF1_-PPH21-T_PGK1_ |  | pRS416-P_TEF1_-PPH21-T_PGK1_ |  |  |
| pRS426-P_TEF1_-BMH1-T_PGK1_ |  | pRS416-P_TEF1_-BMH1-T_PGK1_ |  |  |
| pRS426-P_TEF1_-QCR6-T_PGK1_ |  | pRS416-P_TEF1_-QCR6-T_PGK1_ |  |  |
| pRS414-P_TEF1_-CRS5-T_PGK1_ |  | pRS416-P_TEF1_-CRS5-T_PGK1_ | pRS414 by *XhoI*/*BamHI* |  |
| pRS414-P_TEF1_-CUP1-T_PGK1_ |  | pRS416-P_TEF1_-CUP1-T_PGK1_ |  |  |

**Supplementary Table 3. List of primers used in this study**

| **Primer name** | **Primer sequence (5’→3’)** |
| --- | --- |
| TEF1p For | GGTACCGGGCCCCCCCTCGAAGCTTCAAAATGTTTCTACTCCTTTTTTAC |
| PGK1t Rev | CGCTCTAGAACTAGTGGATCCAGGAAGAATACACTATACTGGATC |
| LEU2-homology For | GTGTAGAATTGCAGATTCCCTTTTATGGATTCCTAAATCCCAGGAAGAATACACTATACTGGATC |
| LEU2-homology Rev | TCGACTACGTCGTAAGGCCG |
| ACT1-RT-PCR For | CGTCTGGATTGGTGGTTCTA |
| ACT1-RT-PCR Rev | GTGGTGAACGATAGATGGAC |
| CAD1-RT-PCR For | CGCACACTCAAGAGAATGAGA |
| CAD1-RT-PCR Rev | ACGGCACCTGTTCGATTT |
| CRS5-RT-PCR For | CTGCCTTCCAAGCTGTTCT |
| CRS5-RT-PCR Rev | GCACGTGGTTTCGCATTT |
| CUP1-RT-PCR For | TCATGTAGCTGCCCAACGG |
| CUP1-RT-PCR Rev | AGAGCAGCATGACTTCTTGGTT |
| NRG1-RT-PCR For | GCGAAAGCAAAGAACAGATCC |
| NRG1-RT-PCR Rev | CCCGATGTAGTGAATCCTCTTG |
| PPH21-RT-PCR For | CATGGGTGATTACGTGGATAGAG |
| PPH21-RT-PCR Rev | CTCGTGATTGCCTCTCAGTATC |
| BMH1-RT-PCR For | ATTGCCACCACAGAGTTACC |
| BMH1-RT-PCR Rev | CAGGCTTTGTCTGGAGAGTTT |
| QCR6-RT-PCR For | GGCAGCAGAAGGAGAAGAAA |
| QCR6-RT-PCR Rev | CTTCCTCATCTTCGTCGTCATC |

**Supplementary Table 4.** **Go Ontology (GO)analysis of identified genes in this study**

| **Gene target** | **Cadmium tolerance** | **Cellular component** | **Biological process** | **Molecular function** |
| --- | --- | --- | --- | --- |
| *CAD1* | 1.856 | Nucleus; cytoplasm | Response to chemical; transcription by RNA polymerase II | DNA binding; DNA-binding transcription factor activity |
| *CRS5* | 1.625 | Cytoplasm | Response to chemical; | Ion binding |
| *CUP1* | 1.442 |  | Response to chemical; response to oxidative stress | Ion binding; oxidoreductase activity |
| *NRG1* | 1.382 | Nucleus | Response to chemical; transcription by RNA polymerase II; pseudohyphal growth; response to osmotic stress | Ion binding; DNA binding; transcription factor binding; DNA-binding transcription factor activity |
| *PPH21* | 1.338 | Nucleus; cytoplasm | Mitotic cell cycle; regulation of translation; regulation of organelle organization; protein dephosphorylation; cytoskeleton organization; cell budding; organelle assembly; vacuole organization | Ion binding; phosphatase activity; hydrolase activity |
| *BMH1* | 1.282 | Nucleus; cytoplasm; plasma membrane | Transcription by RNA polymerase II; mitotic cell cycle; pseudohyphal growth; cell wall organization or biogenesis; protein modification by small protein conjugation or removal; regulation of protein modification process; meiotic cell cycle; carbohydrate metabolic process; sporulation; cellular response to DNA damage stimulus; generation of precursor metabolites and energy; organelle fission; regulation of cell cycle | DNA binding; transcription factor binding |
| *QCR6* | 1.093 | Nucleus; membrane; mitochondrion; mitochondrial envelope | Cellular respiration | oxidoreductase activity |
| ^a^ Cadmium tolerance was calculated to compare the performance in cadmium resistance of different strains in the presence of cadmium nitrate. The tolerance fold change for specific gene was calculated using equation (1) as described in Materials and Methods. | | | | |

**Supplementary Table 5. Protein function of identified genes and speculative molecular mechanisms for cadmium tolerance**

| **Gene target** | **Protein function** | **Speculative molecular mechanisms** | **Reference** |
| --- | --- | --- | --- |
| *CAD1* | AP-1-like basic leucine zipper (bZIP) transcriptional activator | Upregulating FRM2 for oxidative stress attenuation; activating gene expression of SLT2, RLM1 and CHS1 for cell-wall maintenance | (Azevedo et al., 2007; Mazzola et al., 2015) |
| *CRS5* | Metallothionein-like protein | Cadmium binding | (Pagani et al., 2007) |
| *CUP1* | Metallothionein | Cadmium binding | (Ecker et al., 1986; Jeyaprakash et al., 1991) |
| *NRG1* | Transcriptional regulator | Transcriptional repression of TAT1, whose deletion increased cadmium tolerance | (Ruotolo et al., 2008) |
| *PPH21* | Catalytic subunit of protein phosphatase 2A (PP2A) | Mediating cadmium-induced repression of class I transcription by targeting the formation/dissociation of the polI-Rxn3 complex; inactivation of Eerk1/2 and JNK; regulation of MT expression through dephosphorylation of MTF-1 | (Chen et al., 2008; Zhou et al., 2013; Chen et al., 2014) |
| *BMH1* | 14-3-3 protein, major isoform | Preventing from stress-induced apoptosis | (Clapp et al., 2012) |
| *QCR6* | Subunit 6 of the ubiquinol cytochrome-c reductase complex | Regulation of cadmium-induced ROS production | (Wang et al., 2004) |


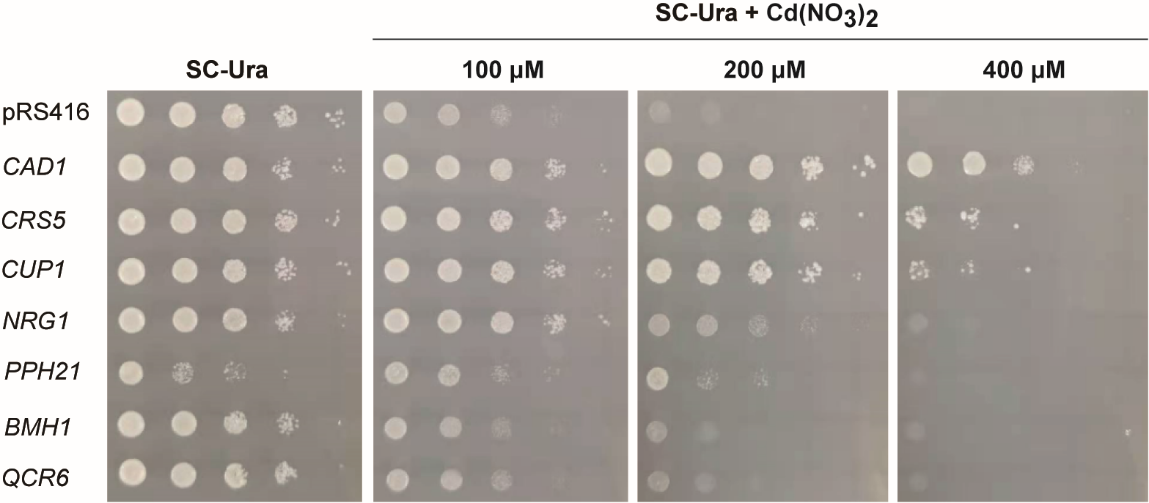


**Supplementary Figure 1.** Cadmium tolerance of yeast strains with single-gene overexpression from pRS416-based plasmids by spot assay. Yeast cultures were serially diluted, spotted, and cultivated for 2-3 days on agar plates without or with 100 μM, 200 μM or 400 μM cadmium.


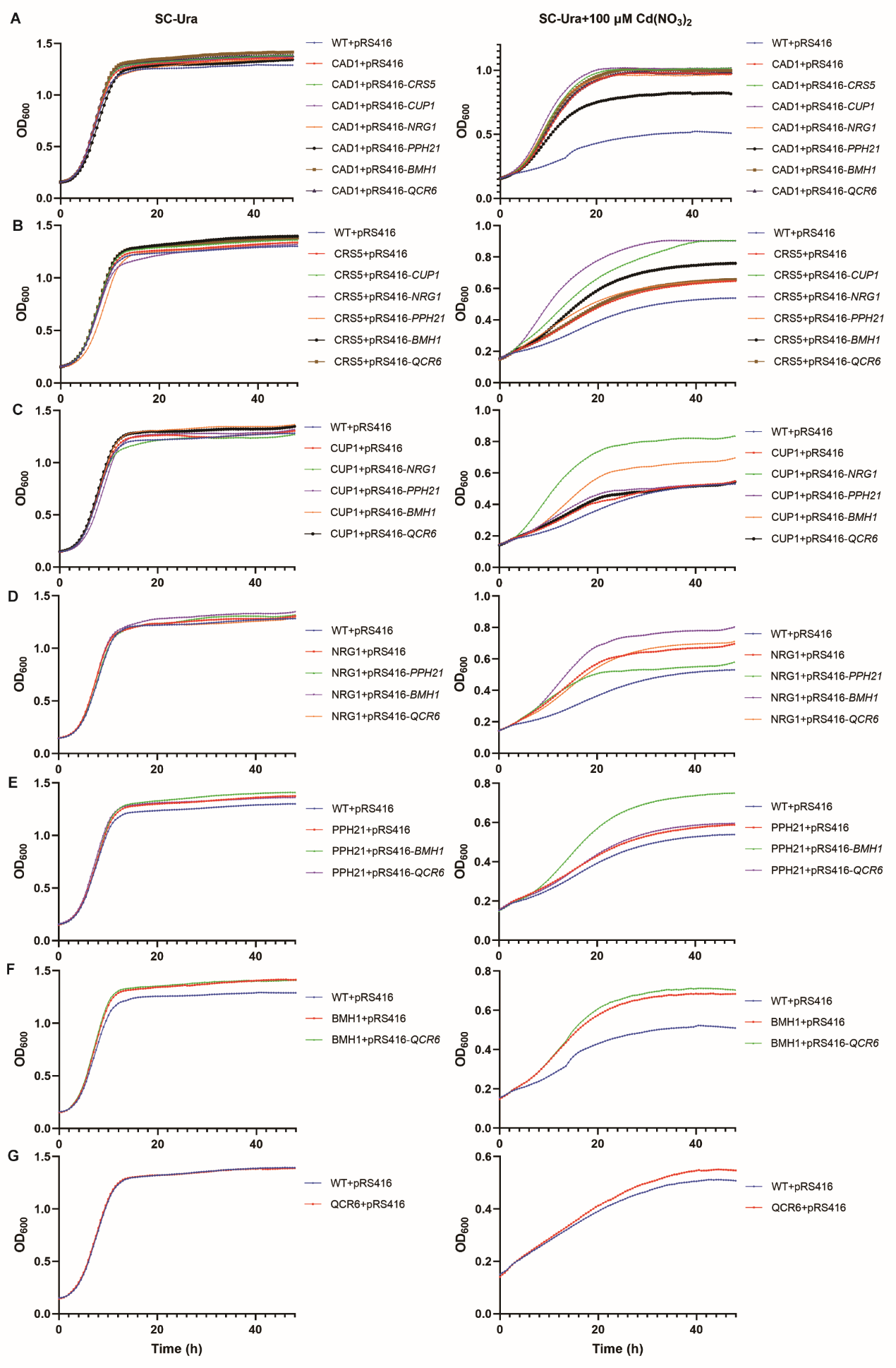


**Supplementary Figure 2.** Growth curve of engineered strains. Wild-type and modified strains with additional genome-integrated copies of *CAD1* (**A**), *CRS5* (**B**), *CUP1* (**C**), *NRG1* (**D**), *PPH21* (**E**), *BMH1* (**F**) and *QCR6* (**G**) were transformed with indicated plasmids. Cellular growth was monitored by measuring OD_600_ every 30 min at 30 ℃ for 48 h in SC-Ura with or without 100 μM cadmium.


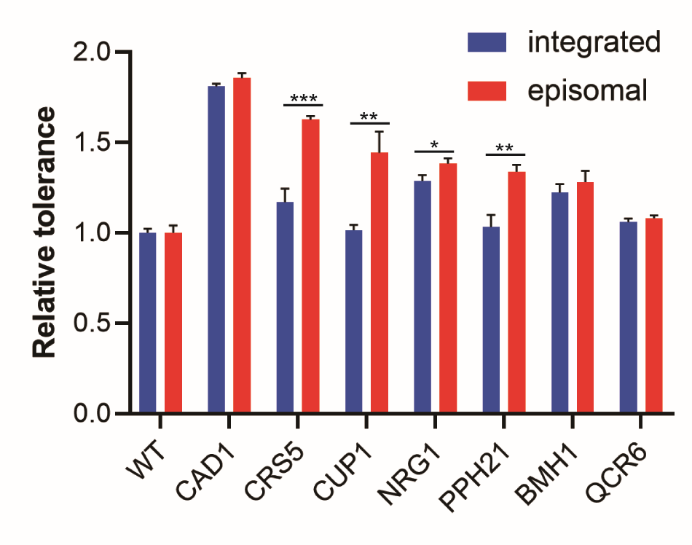


**Supplementary Figure 3.** Relative cadmium tolerance due to single-gene overexpression by genomic integration and episomal plasmid. Cadmium tolerance was calculated as described in Materials and Methods. Values are means and standard derivations (n=3). *, P<0.05; **, P<0.01; ***, P<0.001.


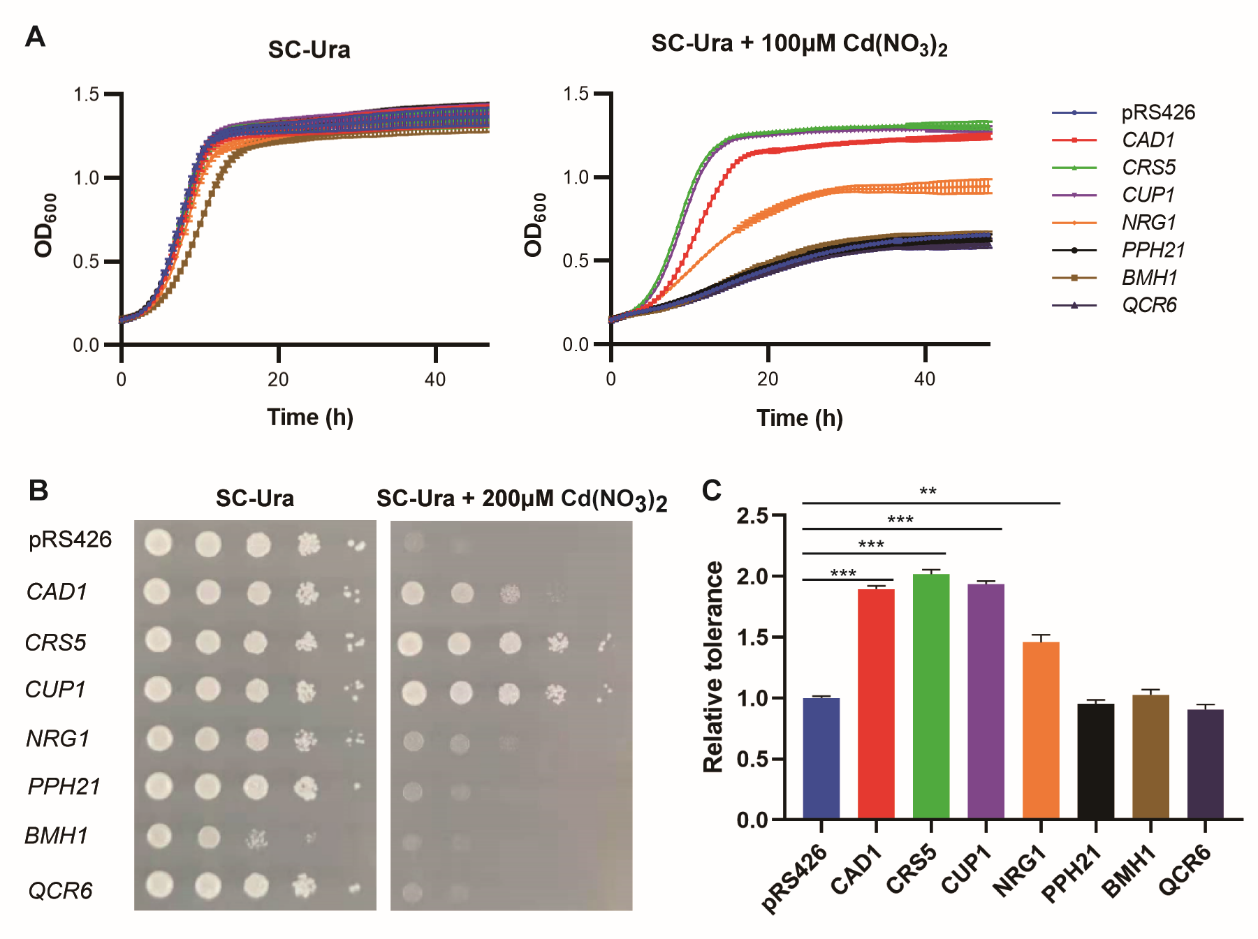


**Supplementary Figure 4.** Cadmium tolerance of yeast strains with single-gene overexpression from multicopy pRS426-based plasmids. **(A)** Time-course of cellular growth in liquid media. The time-course of OD_600_ was collected at 30 min intervals for 48 h with or without 100 μM cadmium. Error bars indicate standard deviations of three biological replicates. **(B)** Spot assay on agar media. Yeast cultures were serially diluted, spotted, and cultivated for 2-3 days on agar plates with or without 200 μM cadmium. **(C)** Relative cadmium tolerance due to gene overexpression from pRS426-based plasmids. The cadmium tolerance was calculated as described in Materials and Methods. Values are means and standard derivations (n=3). *, P<0.05; **, P<0.01; ***, P<0.001.


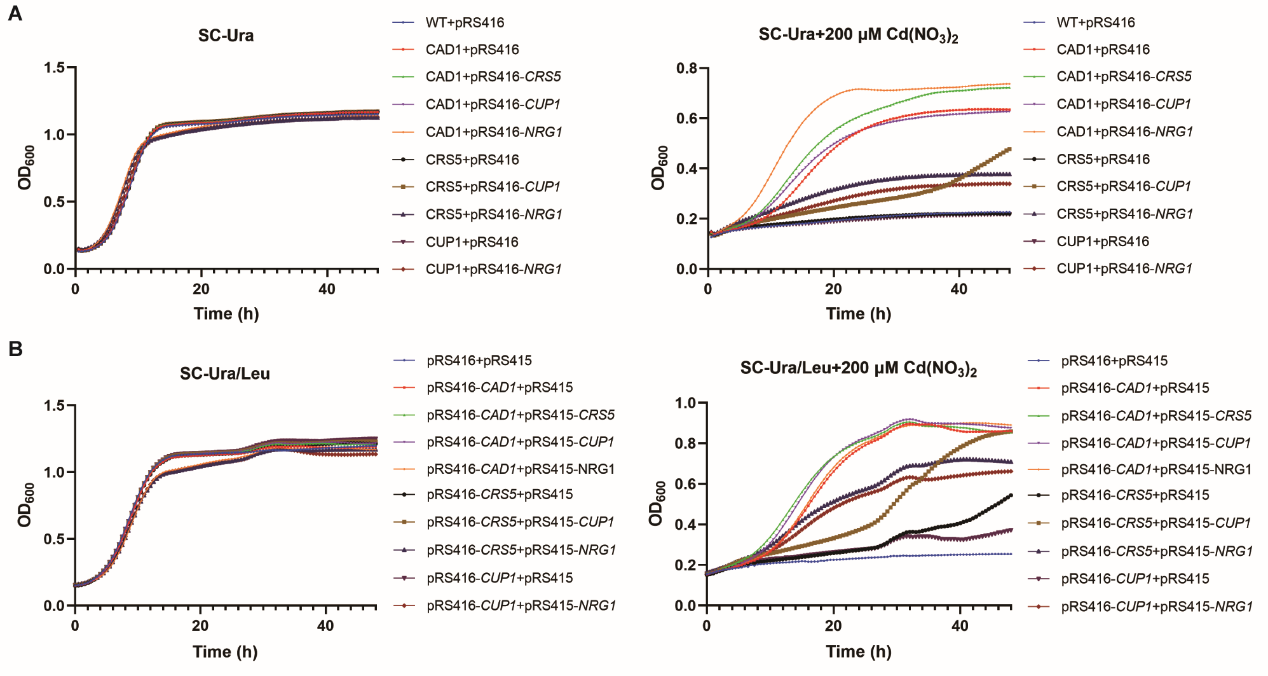


**Supplementary Figure 5.** Growth curve of yeast strains harboring two overexpression cassettes via integration-plasmid and plasmid-plasmid formats. Strains with a modified (**A**) and the wild-type (**B**) genome were transformed with indicated plasmids and seeded into SC-Ura or SC-Ura/Leu medium with or without 200 μM cadmium. Cellular growth was monitored by measuring OD_600_ every 30 min at 30 ℃ for 48 h.


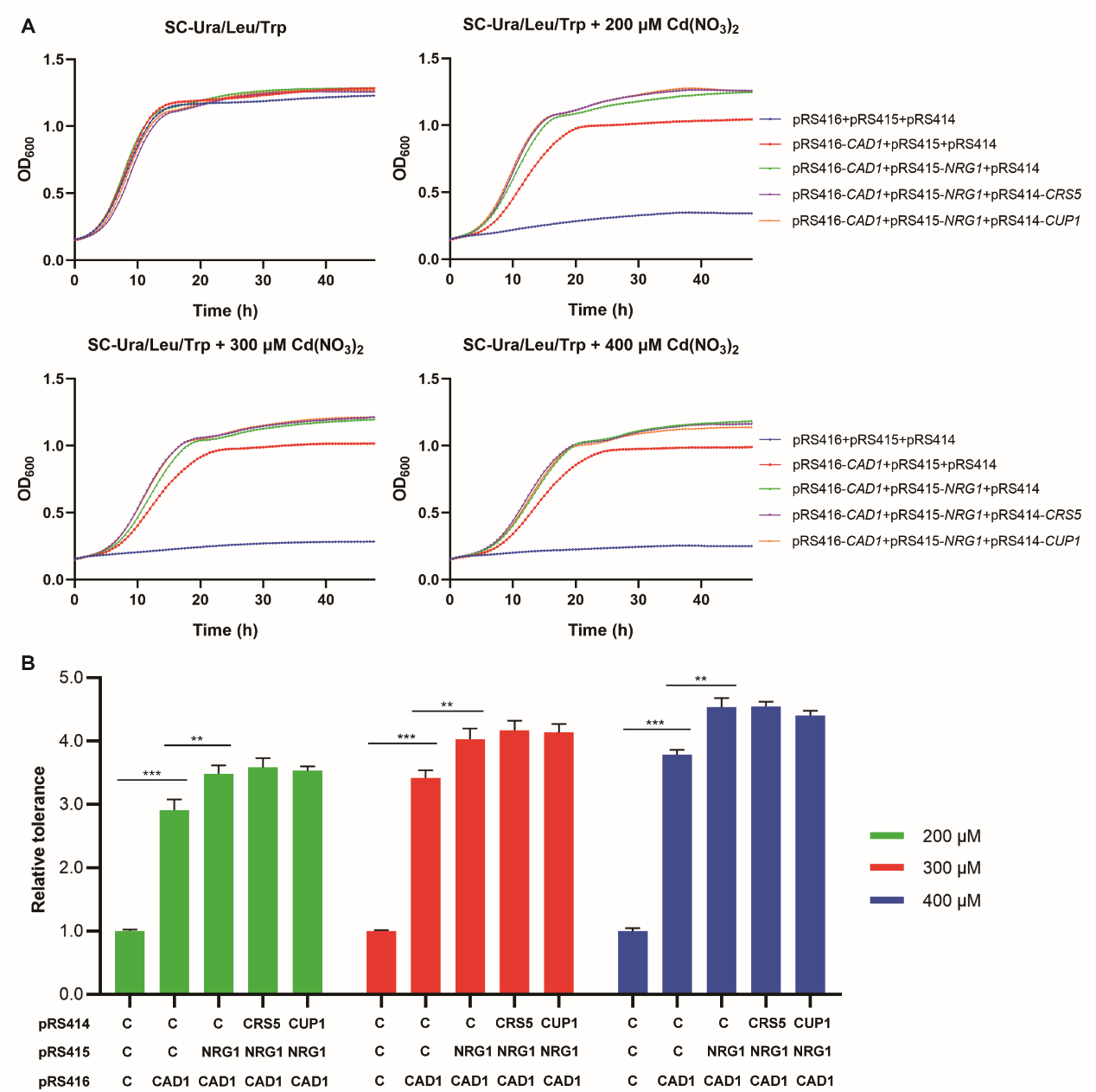


**Supplementary Figure 6**. Growth curve of wild-type strains co-transformed with three plasmids. (**A**) Wild-type strains were transformed with indicated plasmids and seeded into SC-Ura/Leu/Trp medium without or with 200 μM, 300 μM or 400 μM cadmium. Cellular growth was monitored by measuring OD_600_ every 30 min at 30 ℃ for 48 h. (**B**) Relative cadmium tolerance. Values are means and standard derivations (n=3). *, P<0.05; **, P<0.01; ***, P<0.001.

**Supplementary references**

Azevedo D., Nascimento L., Labarre J., Toledano M. B., and Rodrigues-Pousada C. (2007). The *S. cerevisiae* Yap1 and Yap2 transcription factors share a common cadmium-sensing domain. *FEBS Lett* 581, 187-95. doi: 10.1016/j.febslet.2006.11.083

Chen L., Liu L., and Huang S. L. (2008). Cadmium activates the mitogen-activated protein kinase (MAPK) pathway via induction of reactive oxygen species and inhibition of protein phosphatases 2A and 5. *Free Radical Bio Med* 45, 1035-1044. doi: 10.1016/j.freeradbiomed.2008.07.011

Chen L. P., Ma L., Bai Q., Zhu X. N., Zhang J. M., Wei Q., et al. (2014). Heavy Metal-induced Metallothionein Expression Is Regulated by Specific Protein Phosphatase 2A Complexes. *J Biol Chem* 289, 22413-22426. doi: 10.1074/jbc.M114.548677

Clapp C., Portt L., Khoury C., Sheibani S., Norman G., Ebner P., et al. (2012). 14-3-3 Protects against stress-induced apoptosis. *Cell Death Dis* 3. doi: 10.1038/cddis.2012.90

Ecker D. J., Butt T. R., Sternberg E. J., Neeper M. P., Debouck C., Gorman J. A., et al. (1986). Yeast metallothionein function in metal ion detoxification. *J Biol Chem* 261, 16895-900. doi: 10.1016/S0021-9258(19)75973-0

Jeyaprakash A., Welch J. W., and Fogel S. (1991). Multicopy CUP1 plasmids enhance cadmium and copper resistance levels in yeast. *Mol Gen Genet* 225, 363-8. doi: 10.1007/BF00261675

Mazzola D., Pimentel C., Caetano S., Amaral C., Menezes R., Santos C. N., et al. (2015). Inhibition of Yap2 activity by MAPKAP kinase Rck1 affects yeast tolerance to cadmium. *FEBS Lett* 589, 2841-9. doi: 10.1016/j.febslet.2015.07.049

Pagani A., Villarreal L., Capdevila M., and Atrian S. (2007). The *Saccharomyces cerevisiae* Crs5 Metallothionein metal-binding abilities and its role in the response to zinc overload. *Molecular Microbiology* 63, 256-269. doi: 10.1111/j.1365-2958.2006.05510.x

Ruotolo R., Marchini G., and Ottonello S. (2008). Membrane transporters and protein traffic networks differentially affecting metal tolerance: a genomic phenotyping study in yeast. *Genome Biol* 9. doi: 10.1186/gb-2008-9-4-r67

Si T., Luo Y., Bao Z., and Zhao H. (2015). RNAi-assisted genome evolution in *Saccharomyces cerevisiae* for complex phenotype engineering. *ACS Synth Biol* 4, 283-91. doi: 10.1021/sb500074a

Wang Y., Fang J., Leonard S. S., and Rao K. M. (2004). Cadmium inhibits the electron transfer chain and induces reactive oxygen species. *Free Radic Biol Med* 36, 1434-43. doi: 10.1016/j.freeradbiomed.2004.03.010

Zhou L., Le Roux G., Ducrot C., Chedin S., Labarre J., Riva M., et al. (2013). Repression of class I transcription by cadmium is mediated by the protein phosphatase 2A. *Nucleic Acids Res* 41, 6087-97. doi: 10.1093/nar/gkt335
